# Dietary fat and risk of breast cancer

**DOI:** 10.1186/1477-7819-3-45

**Published:** 2005-07-18

**Authors:** Bhaskarapillai Binukumar, Aleyamma Mathew

**Affiliations:** 1 Division of Epidemiology and Clinical Research, Regional Cancer Centre, Thiruvananthapuram – 695011 Kerala, India

## Abstract

**Background:**

Breast cancer is one of the major public health problems among women worldwide. A number of epidemiological studies have been carried out to find the role of dietary fat and the risk of breast cancer. The main objective of the present communication is to summarize the evidence from various case-control and cohort studies on the consumption of fat and its subtypes and their effect on the development of breast cancer.

**Methods:**

A Pubmed search for literature on the consumption of dietary fat and risk of breast cancer published from January 1990 through December 2003 was carried out.

**Results:**

Increased consumption of total fat and saturated fat were found to be positively associated with the development of breast cancer. Even though an equivocal association was observed for the consumption of total monounsaturated fatty acids (MUFA) and the risk of breast cancer, there exists an inverse association in the case of oleic acid, the most abundant MUFA. A moderate inverse association between consumption of n-3 fatty acids and breast cancer risk and a moderate positive association between n-6 fatty acids and breast cancer risk were observed.

**Conclusion:**

Even though all epidemiological studies do not provide a strong positive association between the consumption of certain types of dietary fat and breast cancer risk, at least a moderate association does seem to exist and this has a number of implications in view of the fact that breast cancer is an increasing public health concern.

## Background

Breast cancer is the most common malignancy amongst women worldwide, constituting 10% of all cancers. It is estimated that more than 1.1 million new breast cancer cases occur worldwide annually representing over twenty percent of malignancies in women [1]. Incidence rates of breast cancer are approximately 90–130 per 100,000 women in developed countries and those in developing countries are approximately ten to sixty per 100,000 women [1]. Several studies have been carried out to identify the risk factors for developing breast cancer. Studies of reproductive factors suggest that nulliparity and late age at first childbirth are the most consistent risk factors associated with breast cancer [2]. Certain dietary factors such as a higher intake of fat and meat also seem to increase the risk of breast cancer [3,4]. Fat is a macronutrient and is considered to be a major source of calories or energy. Although some fat in the diet is necessary, too much of fat can lead to heart diseases, cancers, obesity and other health problems. Numerous studies in women, using different study designs and in different geographical areas have been carried out in order to establish the relationship of dietary fat to breast cancer risk.

The objective of the present communication is to summarize evidence from various case-control and cohort studies on the consumption of dietary fat and its sub-types [saturated, monounsaturated (MUFA) and polyunsaturated (PUFA) fatty acids] and their effect on the development of breast cancer. The major sources of saturated fats are meat, poultry, dairy products, eggs and some plant foods such as coconut, coconut oil and palm oil [5]. High concentrations of MUFA are found in vegetable oils and their traces are also found in meat. Oleic acid, the most abundant MUFA, is found in animal and vegetable oils. Oleic acid is the major component of olive oil. PUFAs are classified into two families – n-3 PUFAs and n-6 PUFAs. PUFAs are mostly found in plants, fish and seafoods. Rich sources of n-3 PUFA include mackerel, salmon, and albacore tuna [6]. Types of n-3 fatty acids include eicosapentaenoic acid (EPA; 20:5 n-3), docosahexaenoic acid (DHA; 22:6 n-3), α-linolenic acid (18:3 n-3) and stearidonic acid (18:4 n-3). EPA and DHA are present in fatty fish although it can be obtained from enriched eggs [7]. N-6 fatty acids are found in high concentration in corn oil, safflower oil, soybean oil, sunflower oil and cottonseed oil. Types of n-6 fatty acids include linoleic acid (18:2 n-6), arachidonic acid (20:4 n-6), docosapentaenoic acid (DPA; 22:5 n-6) and γ-linolenic acid (18:3 n-6) [8].

## Methods

A Pubmed search of the literature was carried out covering studies conducted over a period of fourteen years (from 1990 to 2003) using keywords "*dietary fat" and "risk" and "breast cancer"*. The information on study design, country, data collection method, study period, sample size, study subjects, confounding variables, partition values used for comparisons, odds ratio/relative risk and trend p-value (if available) were obtained from the articles. Only case-control and cohort studies conducted among women were considered for the present review. Reports from symposia, genetic studies, survival/mortality studies of breast cancer patients and all studies associated with breast cancer other than dietary fat in women were excluded from the present review.

## Total dietary fat

### Case-control studies

Of the seventeen case-control studies [6,9-24] that investigated the relation of total dietary fat with risk of breast cancer, seven studies reported a significant positive association between total fat intake and risk of breast cancer [10,11,17-19,22,24]. The odds ratios (OR) of these studies ranged from 1.45 to 8.47 in the highest category of total fat consumption. Significant dose-response relationships between the increased consumption of total fat and breast cancer risk were observed in four studies [11,18,22,24]. Increased risk with border line significance was found for the intake of fat meat in one study [OR = 1.18 (95% CI: 1.0–1.5) in the highest quartile of fat meat intake] [17]. Non-significant increased risks were reported in six studies [6,14,16,20,21,23]. Significant reduced risk with a dose-response relationship was observed in one study [12]. Three studies have shown no association [9,13,15] (Additional file 1).

### Cohort studies

Of the twenty cohort studies, which investigated the relationship between total dietary fat intake and breast cancer risk, ten were based exclusively on post-menopausal women [25-34]. Five studies showed significant positive associations with dose-response relationships between total fat and breast cancer risk [25,26,31,34,35]. The relative risks for these studies ranged from 1.15 to 3.47 in the highest level of total fat consumption. Non-significant positive associations between total fat and the risk of breast cancer were observed in nine studies [30,32,33,36-41]. Six studies found no association between the fat intake and the risk of breast cancer [27-29,42-44] (Additional file 2).

## Components of fat

### Saturated fat

#### Case-control studies

Four studies out of the seventeen reviewed here found significant positive associations with dose-response relationships between saturated fat and breast cancer [9,18,19,45]. The odds ratios for these studies ranged from 1.90 to 2.38. Nine studies showed non-significant increase in associations of saturated fat with the development of breast cancer [12,14,16,21,23,46-48,66]. Four studies reported no association between saturated fat and breast cancer risk [6,15,49,50] (Additional file 1).

#### Cohort studies

Among the seventeen cohort studies, three reported positive associations with significant dose-response relationships between the consumption of saturated fat and the risk of breast cancer [26,36,41]. The relative risks for these studies ranged from 1.13 to 1.47 in the highest level of saturated fat consumption. Non-significant increased associations for breast cancer were found in eight studies [30-33,37,38,40,51]. Six studies found no associations between the consumption of saturated fat and the risk of breast cancer [27,28,34,42,43,52] (Additional file 2).

### Monounsaturated fat -Oleic acid

#### Case-control studies

Two studies of increased consumption of oleic acid (the most abundant MUFA) [12,57] and four studies of increased consumption of olive oil (a major component of oleic acid) [48,54-56] reported reduced risks of breast cancer. No association was observed in one study for the consumption of oleic acid [53] (Additional file 1).

#### Cohort studies

Some cohort studies found that increased consumption of oleic acid reduced the risk of breast cancer but the associations were non-significant [32,33,43]. However, other studies have shown no association [27,40] (Additional file 2).

### Total monounsaturated fat

#### Case-control studies

Contrary to the studies on oleic acid consumption, three of the twelve case-control studies reported positive associations with a dose-response relationship between increased consumption of total MUFA and the risk of breast cancer [16,18,23]. The odds ratios for these studies ranged from 1.04 to 1.89 in the highest level of MUFA consumption. Non-significant increased associations for higher consumption of MUFA were found in three studies [6,21,23]. The rest of the studies reviewed reported no association between MUFA consumption and subsequent risk of breast cancer [14,15,46,48-50] (Additional file 1).

#### Cohort studies

Of the twelve studies that have been reviewed, three showed significant positive associations with dose-response relationships for increased intake of MUFA and the risk of breast cancer [34,37,38]. The relative risks for these studies ranged from 1.72 to 2.01 in the highest level of MUFA consumption. Five studies reported non-significant increased breast cancer risk [28,30,31,36,51]. Three studies showed inverse associations with significant dose-response relationships between the higher intake of MUFA and the risk of breast cancer [33,42,52]. The relative risks of these studies varied from 0.61 to 0.94. No significant association between the consumption of MUFA and breast cancer was found in one study [41] (Additional file 2).

### Total polyunsaturated fat

#### Case-control studies

Six studies out of fourteen reported a significantly reduced breast cancer risk with increased PUFA consumption [12,15,45,48,57,66]. The odds ratios for these studies ranged from 0.39 to 0.70 in the highest category of PUFA consumption. Decreased breast cancer risk with significant dose response patterns was reported for high consumption of PUFA in three studies [12,15,57]. No associations were found between PUFA intake and risk of breast cancer in eight studies [6,14,16,18,21,23,46,49] (Additional file 1).

#### Cohort studies

Contrary to the results based on case-control studies, three out of eleven cohort studies reported significant positive associations with dose-response relationship between the increased consumption of PUFA and the risk of breast cancer [30,34,52]. The relative risks of these studies ranged from 1.49 to 3.02. Four studies reported non-significant increased risks associated with increased PUFA consumption [28,31,36,38]. Four studies reported no association between the consumption of PUFA and risk of breast cancer [33,41,42,51] (Additional file 2).

### n-3 fatty acids

#### Case-control studies

One study of n-3 fatty acids [58] and another study of DHA reported significantly reduced risks for breast cancer [59] (Additional file 1). A significantly reduced breast cancer risk with a dose-response relationship was observed in one study for the increased consumption of DHA (n-3 fatty acid) [59]. Two studies of consumption of alpha-linolenic acid showed inverse association with a significant dose-response relationship [50,59]. One study of EPA (n-3 fatty acid) showed an inverse association [58]. No association was observed in other studies for the increased consumption of n-3 fatty acids [6,16,53,55] with the development of breast cancer (Additional file 1).

#### Cohort studies

Significantly reduced risk of breast cancer was observed in one study for increased consumption of n-3 fatty acids (RR = 0.72 in the highest quartile of intake) [28]. Contrary to the above result, a Swedish cohort study among post-menopausal women reported a significantly increased breast cancer risk with dose-response relationship [34]. The other studies showed no associations for breast cancer incidence on the consumption of n-3 fatty acids [36,51], DHA [33] and EPA [33,51] (Additional file 2).

### n-6 fatty acids

#### Case-control studies

One study from France of linoleic acid (n-6 fatty acid) consumption reported a highly significant increased breast cancer risk (OR = 2.31 in the highest category of consumption of linoleic acid) [59]. Two studies of n-6 fatty acids reported non-significant increased breast cancer risks [55,58]. No associations were observed for the consumption of n-6 fatty acids [50,53,59] with the development of breast cancer (Additional file 1).

#### Cohort studies

One study conducted in Sweden among postmenopausal women reported a significantly increased breast cancer risk with dose-response relationship on the consumption of n-6 fatty acids [OR = 3.02 (95% CI: 1.78–5.13) in the highest quintile] [34]. Non-significant increased breast cancer risks were observed with increased consumption of n-6 fatty acids [28] and linoleic acid (n-6 fatty acid) [31,33]. A few studies found no risk of breast cancer with the consumption of linoleic acid [27,32,40,43] and arachidonic acid (n-6 fatty acid) [33] (Additional file 2).

## Discussion

Most of the case-control studies discussed here have shown increased breast cancer risk with increased total fat consumption. However, results of cohort studies did not offer the same degree of consistent support.

Case-control and cohort studies share some strengths and limitations. The primary advantages are the ability to measure diet and potential confounding factors relatively accurately and uniformly at the individual level in both the types of study designs. Also, the subjects in most of the studies were generally drawn from a relatively uniform population. Despite these advantages, there remains a number of limitations common to both case-control and cohort studies.

As fat intake is estimated by food frequency questionnaire (FFQ) in most of the studies discussed in the present review, there remains a certain amount of measurement error in such procedures. Non-differential random measurement error (i.e. independent of case or non-case status) in estimated fat intake might bias estimated risks toward the null and concurrent measurement error in potential confounding variables (e.g., other sources of energy intake) could bias results in either direction. Only a few studies mentioned about validated FFQ [6,14,24,28,33,53,66]. Non-validated questionnaire for collecting the dietary information might also affect the reliability of study results. Another important limitation identified by the present review is that most of the studies are generally conducted in a relatively homogenous population; hence the range of fat intake and the power of the study to detect a true association is restricted. Due to the lack of a definitive biologic mechanism and the lack of information on the relevant timing of exposure, it is possible that case-control and cohort studies are measuring diet at the wrong point in time; this again might attenuate the observed risk in any particular study.

The main contrast between case-control and cohort studies lies in their differing potential for bias and in the resources required. Selection bias would occur if participation in a case-control study was correlated with fat intake and if cases and controls have different participation rates. In many of the case-control studies discussed here, particularly those conducted in highly industrialized populations such as the United States, there is often a substantial refusal to participate, particularly among control subjects, and it is possible that there is preferential participation by health-conscious individuals, who might have a relatively low fat intake. Such a phenomenon might introduce an artificial positive association between fat intake and breast cancer risk. Recall bias might occur since cases are interviewed after diagnosis and might report their past diet differently to control subjects. In particular, they could report their diets more accurately through better motivation, or conversely, they could report an over inflated fat consumption if aware of the postulated association between fat intake and breast cancer risk.

Since there are many limitations associated with case-control studies, the results from the cohort studies can be considered more authentic. Cohort studies assessing the relationship between total dietary fat and risk of breast cancer have been used since the late 1980's as an alternative to the inconsistent findings of the case-control studies. These are normally considered free of the most common biases that potentially affect case-control studies such as selection and information bias.

Even though most of the cohort studies failed to find an association between total dietary fat intake and breast cancer risk, some studies reported a significant positive association with dose-response relationship [25,26,31,34,35]. The failure of cohort studies to show a consistent and a strong relationship may be attributed to the difficulty in collecting accurate dietary information. The methodological limitations associated with the study designs and inaccuracies in the measurement of fat may have obscured any relationship between fat and breast cancer that might exist.

When we consider the components of fat, saturated fat, the unhealthiest out of all the types of fat, when consumed in large amounts increases the risk of breast cancer. In the present review, several case-control studies have shown positive association with breast cancer risk [9,18,19,45]. However, there are only a few cohort studies [26,36,41] reported statistically significant increased breast cancer risk with increased saturated fat consumption.

Monounsaturated fats (MUFA) are considered as 'non-essential' because they can be synthesized within our bodies, from foods and oils such as oleic acid and olive oil. Even though a few studies showed a positive correlation between MUFA and breast cancer [16,18,34,37], most of the studies reviewed here have shown that oleic acid including olive oil has an inverse relationship with breast cancer [12,16,18,33,34,37]. Many previous epidemiological studies have pointed out the preventive properties of olive oil due to its high content of oleic acid [56,60,61].

In the present review on polyunsaturated fatty acids, we focused mainly on n-6 and n-3 families. Various studies have analyzed the possible relation of (n-3)/(n-6) PUFA ratio and the possible role that it may play in the risk of breast cancer [6,58]. Most of the studies reviewed have shown that a higher (n-3)/(n-6) PUFA ratio may reduce the risk of breast cancer [6,58,59,62]. Some studies, however, found results which suggests that high intake of monounsaturated fatty acids, n-3 fatty acids and n-6 fatty acids were related to lower risk [63] and the same study, which primarily investigated the role of recall bias, found no relation between PUFA and breast cancer risk.

Boyd and others [4] carried out a meta analysis of all published studies up to July 2003. They found that 14 cohort and 31 case-control studies gave similar results and suggested that total and saturated fat intake are positively associated with increased risk of breast cancer. Saadatian-Elahi and others [64] analyzed 3 cohort and 7 case-control studies including 2,031 cases and 2,334 controls and concluded that n-3 PUFA had a protective effect and total MUFA and oleic acid levels were significantly associated with an increase of breast cancer risk. In a pooled analysis, Hunter and others [65] gathered information about 4,980 cases from studies including 337,819 women and found that there was no evidence of association between total dietary fat intake and the risk of breast cancer. The meta-analytical studies arrived at a conclusion similar to the present review and suggest that certain dietary fats are associated with breast cancer risk. Only Hunter *et al*, [65] have differed in that they found no association. Prentice and Sheppard [83] have, in fact, concluded that 50% reduction of dietary fat from current USA consumption levels would result in a 2.5 fold reduced risk of breast cancer among postmenopausal women.

Previously published review papers reported increased risk of breast cancer with the increased consumption of total dietary fat [68,69,71-75]; saturated fat [69,76-79] and moderately decreased association with oleic acid and monounsaturated fatty acids [67,70,80,81]. These results are consistent with our present review except for an equivocal association observed in the case of monounsaturated fatty acids. A few reviews have shown no association of various types of fat with the etiology of breast cancer [76,82]. Previous epidemiologic evidence provides little support for any important relation between intake of either linoleic acid (n-6 fatty acid) or n-3 fatty acids and risk of breast cancer [67] as against a moderate inverse association between the increased consumption of n-3 fatty acids and the risk of breast cancer and a moderate positive association between the increased consumption of n-6 fatty acids and the risk of breast cancer.

## Conclusion

Considering the totality of epidemiologic evidence, it is reasonable to conclude that a positive association exists between total dietary fat intake and breast cancer risk, even though all epidemiological studies do not provide a strong association between consumption of dietary fat and breast cancer risk. Also, increased consumption of saturated fat is positively associated with the development of breast cancer. There exists an inverse association of breast cancer risk with the consumption of oleic acid. A moderate inverse association between breast cancer risk and the consumption of n-3 fatty acids and a moderate positive association between n-6 fatty acids and breast cancer risk were also observed. These have lot of implications in view of the fact that breast cancer is a growing public health problem.

## Competing interests

The present review paper will be quite useful for surgeons, clinicians, epidemiologists and other public health professionals particularly working in the filed of cancer.

## Authors' contributions

BB – collected the literature, summarized the results in the form of tables and wrote the paper

AM: provided technical guidance for writing the paper and edited the paper

## Funding sources

Nil

## Supplementary Material

Additional File 1 Table showing case control studies and risk of breast cancer. Click here for file

Additional File 2 Table showing cohort studies and risk of breast cancer. Click here for file
