# Supplementary material for: Dietary fat and risk of breast cancer
Source: World J Surg Oncol. 2005 Jul 18;3:45. doi: 10.1186/1477-7819-3-45 (PMC1199629; doi:10.1186/1477-7819-3-45)
Supplement: Additional File 1 — Table showing case control studies and risk of breast cancer. [file 1477-7819-3-45-S1.doc]

# Additional file 1. Case control studies on dietary fat and risk of breast cancer

|  | **RR**^a^ (Highest vs lowest category) | | | | | | | Confounding factors |
| --- | --- | --- | --- | --- | --- | --- | --- | --- |
|  |  | **Total fat** | **SFA^b^** | **MUFA^c^** | **Total PUFA^d^** | **n-3 PUFA** | **n-6 PUFA** |  |
| Author  Year  Country  Data  nb cases/nb controls  Menopausal status | Goodstine et al (2003)  1994-‘97  USA  FFQ^e^  565 cases of 554 controls  Both pre- & postmenopausal | 1.08  (0.64-1.84)  p=0.73 | 0.97  (0.59-1.58)  p=0.83 | 1.17  (0.70-1.95)  p=0.55 | 1.06  (0.68-1.64)  p=0.82 | EPA^f^=0.94  (0.66-1.34)  p=0.70 | ---- | Age, age at menarche, menopausal status, age at first childbirth, number of live births, BMI^h^, lactation history, race, family history of BC^i^ and income |
|  |  |  |  |  |  | DHA^g^ =1.00  (0.70-1.44)  p=0.92 |  |  |
| Author  Year  Country  Data  nb cases/nb controls  Menopausal status | Nkondjock et al (2003)  1989-‘93  Canada  FFQ  414 cases & 429 controls  Both | ---- | ---- | *Oleic acid:*  0.97  (0.65-1.44)  p=0.58 | ---- | α-LA^j^=1.27  (0.85-1.89)  p=0.28 | LA^k^=0.90  (0.61-1.34)  p=0.70 | Age at first FTP^n^, history of BC in first degree relatives, history of BBD^o^, number of FTPs, smoking, marital status and total energy intake |
|  |  |  |  |  |  | EPA=1.23  (0.82-1.83)  p=0.48 | DPA^ℓ^=1.33  (0.89-1.99)  p=0.55 |  |
|  |  |  |  |  |  | DHA=0.98  (0.66-1.46)  p=0.55 | AA^m^=0.86  (0.58-1.30)  p=0.72 |  |
| Author  Year  Country  Data  nb cases/nb controls  Menopausal status | Hermann et al (2002)  1992-‘95  Germany  FFQ  355 cases & 838 controls  Premenopausal | 1.18  (0.70-2.0)  p=0.58 | 1.12  (0.62-2.03)  p=0.89 | 1.04  (0.57-1.89)  p=0.80 | 0.88  (0.50-1.56)  p=0.84 | ---- | ---- | Education, duration of breast feeding, first degree family history of BC, number of births, BMI, energy intake, alcohol consumption & non consumption of each specific food group |
| Author  Year  Country  Data  nb cases/nb controls  Menopausal status | Maillard et al (2002)  -----  France  Breast adipose fatty acid levels  241 cases & 88 controls  Both | ---- | ---- | ---- | ---- | α-LA= 0.39^$^  (0.19-0.78)*  p=0.01* | LA=2.31  (1.15-4.67) *  p=0.06 | Age at diagnosis, height, BMI, menopause and menopausal status. |
|  |  |  |  |  |  |  | γ-LA^p^=0.56  (0.26-1.21)  p=0.74 |  |
|  |  |  |  |  |  | DHA=0.31  (0.13-0.75) *  p=0.016* | AA=0.98  (0.42-2.29)  p=0.32 |  |
| Author  **Table 1 (Contd….)**  Year  Country  Data  nb cases/nb controls  Menopausal status | Wakai et al (2000)  1992-‘95  Indonesia  FFQ  226 cases & 452 controls  Pre- & postmarriage | *Pre-marriage:*  8.47  (4.03-17.8)*  p<0.001* | ---- | ----- | ---- | ---- | ---- | BMI, family history of BC, menopausal status, age at menarche, age at first marriage and number of live births |
|  |  | *Post-*  3.48  (1.96-6.17)^*^  p<0.001^*^ |  |  |  |  |  |  |
| Author  Year  Country  Data  nb cases/nb controls  Menopausal status | Sala et al (2000)  1989-‘97  UK  7-DFD^q^  203 cases & 203 controls  Both | 1.24^**^  (0.77-2.00)  p= NS^r^ | ---- | ---- | ---- | ---- | ---- | Menopausal status , parity, HRT^s^, BMI and total energy intake |
| Author  Year  Country  Data  nb cases/nb controls  Menopausal status | Ronco et al (1996)  1994-‘95  Uruguay  FFQ  175 cases & 253 controls  Both | 1.79  (1.01-3.2)*  p=0.07 | 2.38  (1.3-4.33)*  p= 0.01* | ---- | ---- | ---- | ---- | Family history, menopausal status, BMI, total energy and total alcohol |
| Author  Year  Country  Data  nb cases/nb controls  Menopausal status | Franceschi et al (1996)  1991-‘94  Italy  FFQ  2569 cases & 2588 controls  Both | 0.81  p=0.01* | 0.95  p=0.47 | *Oleic acid* : 0.81  p=0.06 | 0.70  p<0.001* | ---- | LA=0.69  p<0.001* | Total energy |
| Author  Year  Country  Data  nb cases/nb controls  Menopausal status | Yuan et al (1995)  1984-‘85  China  FFQ  834 cases & 834controls  Both | 1.2  (0.7-2.0)  p=0.44 | 1.3  (0.6-2.6)  p=0.13 | 1.2  (0.7-2.2) p=0.4 | 1.3  (0.4-4.3)  p=0.35 | ---- | ---- | Total energy, age at menarche, usual cycle length under 25 days, number of FTP’s, duration of nursing years, first used OC’s at age 35+, 61+ kg average weight, had a BBD, BC of female first degree relative & education |
| Author  Year  Country  Data  nb cases/nb controls  Menopausal status | Katsouyanni et al (1995)  1989-‘91  Greece  FFQ  820 cases & 1548 controls  Both | ---- | 0.99  (0.89-1.11) p=0.86 | 0.97  (0.88-1.07)  p=0.53 | 1.05  (0.97-1.13)  p=0.21 | ---- | ---- | Age, place of birth, party, age at first pregnancy, age at menarche, menopausal status, BMI & total energy |
| Author  **Table 1 (Contd….)**  Year  Country  Data  nb cases/nb controls  Menopausal status | London et al (1993)  1986-‘88  USA  FFQ  380 cases & 573 controls  Postmenopausal | 1.5^§^  (0.9-2.6)  p=0.21 | 1.2  (0.7-2.1)  p=0.28 | 1.7  (1.0-2.8)* p=0.12 | 1.0  (0.6-1.7)  p=0.99 | 1.0  (0.6-1.7) p=0.82 | ---- | Age, alcohol, age at first child birth, age at menarche, age at menopause, prior history of breast disease, weight 5 year before the study entry and caloric intake |
| Author  Year  Country  Data  nb cases/nb controls  Menopausal status | Richardson et al (1991)  1983-‘87  France  FFQ  409 cases & 515 controls  Both | 1.60  (1.1-2.2)*  p=0.014* | 1.9  (1.3-2.6)*  p<0.001* | 1.7  (1.2-2.5)*  p=0.002* | 1.2  (0.9-1.7)  p=NS | ---- | ---- | Age, menopausal status, family history of BC, BBD, age at menarche, parity, age at first FTP, BMI & education |
| Author  Year  Country  Data  nb cases/nb controls  Menopausal status | Lee et al (1991)  1986-‘96  Singapore  FFQ  200 cases & 420 controls  Both | 0.75  (0.41-1.36)  p=NS | 0.92  (0.49-1.72)  p=NS | 0.97  (0.51-1.83)  p=NS | 0.39  (0.21-0.70)*  p=0.004* | ---- | ---- | Age, age at first child birth including nulliparous as a category |
| Author  Year  Country  Data  nb cases/nb controls  Menopausal status | Ewertz et al (1990)  1983-‘84  Denmark  FFQ  1474 cases & 1322 controls  Both | 1.45  (1.17-1.8)*  p<0.001* | ---- | ---- | ---- | ---- | ---- | Age at diagnosis and place of residence |

a. Relative risk, b. Saturated fatty acids, c. Monounsaturated fatty acids, d. Polyunsaturated fatty acids, e. Food frequency questionnaire, f. Eicosapentaenoic acid, g. Docosahexaenoic acid, h. Body mass index. i. Breast cancer, j. α – Linolenic acid, k.Linoleic acid, ℓ. Docosapentaenoic acid, m. Arachidonic acid, n. Full term pregnancy, o. Benign breast disease, p. γ – Linolenic acid, q. 7-Day food dairy, r. Not significant, s. Hormone replacement therapy, * Statistically significant at 5% level.

**- The odds ratio estimates for high risk mammographic patterns according to the consumption of red meat.

$- The odds ratios and 95 % confidence intervals were estimated by the adipose tissue fatty acids from the whole populations.

§- Odds ratio estimated by quintile of percentage of specific fatty acids in the subcutaneous adipose tissue of 953 post-menopausal women.

# 
