# Supplementary material for: Dietary fat and risk of breast cancer
Source: World J Surg Oncol. 2005 Jul 18;3:45. doi: 10.1186/1477-7819-3-45 (PMC1199629; doi:10.1186/1477-7819-3-45)
Supplement: Additional File 2 — Table showing cohort studies and risk of breast cancer. [file 1477-7819-3-45-S2.doc]

# Additional file 2. Cohort studies on dietary fat and risk of breast cancer

|  | **RR**^a^ (Highest vs lowest category) | | | | | | | Confounding factors |
| --- | --- | --- | --- | --- | --- | --- | --- | --- |
|  |  | **Total fat** | **SFA**^b^ | **MUFA**^c^ | **Total PUFA**^d^ | **n-3 PUFA** | **n-6 PUFA** |  |
| Author  Year  Country  Data  Cohort size & cases  Menopausal status | Cho et al (2003)  1991-‘99  USA  FFQ^e^  90655 women &714 cases  Premenopausal | 1.25  (0.98-1.59)  p=0.06 | 1.17  (0.91-1.50)  p=0.02* | 1.26  (0.99-1.60)  p=0.06 | 1.06  0.84-1.35  p=0.20 | (0.78-1.31)  p=0.50 | ---- | Age, calendar year of current questionnaire cycle, smoking, height, parity, age at menarche, menopausal status, age at first child birth, OC^f^ use, BMI^g^, family history of BC^h^, history of benign breast disease, alcohol intake, protein and total energy. |
| Author  Year  Country  Data  Cohort size & cases  Menopausal status | Bingham et al (2003)  1993-‘97  UK  7-DFD^i^ & FFQ  13070 women & 168 cases  Postmenopausal | *7-DFD:*  1.15  (1.02-1.31) *  p=0.026^*^ | *7-DFD:*  1.21  (1.07-1.37)*  p=0.026* | ---- | ---- | ---- | ---- | Weight, height, parity, menopausal status & the use of HRT^j^ |
|  |  | FFQ: 1.10  (0.97-1.25)  p=0.14 | FFQ: 1.13  (1.0-1.29)*  p=0.05* |  |  |  |  |  |
| Author    Year  Country  Data  Cohort size & cases  Menopausal status | Gago-Dominiguez et al (2003)  1993-‘98  Singapore  FFQ  35,298 women & 314 cases  Postmenopausal | 0.94  (0.68-1.31)  p=0.95 | 0.73  (0.67-1.26)  p=0.59 | 1.02  (0.73-1.43)  p=0.90 | 1.27  (0.92-1.74)  p=0.46 | 0.72  (0.53-0.98) *  p=0.04* | 1.22  (0.89-1.67)  p=0.45 | Age at baseline interview, year of recruitment, dialect group, education level, daily alcohol consumption, number of live births, age when menstrual periods became regular and family history of BC |
| Author  Year  Country  Data  Cohort size & cases  Menopausal status | Wirfalt et al. (2002)  1991-‘96  Sweden  DHM^k^  12803 women-237 cases & 673 controls  Postmenopausal | 1.51  (0.92-2.49)  p=0.019* | 0.95  (0.57-1.61) p=0.83 | 2.01  (1.19-3.38)*  p=0.001^*^ | 3.02  (1.75-5.21) ^*^  p=0.001^*^ | 1.81  (1.09-2.99)*  p=0.03^*^ | 3.02  (1.78-5.13)*  p=0.0002* | Past food habit change, total energy intake, BMI, height, waist circumference, age at first child birth, current hormone therapy, alcohol use and education |
| Author  **Table 2 (Contd….)**  Year  Country  Data  Cohort size & cases  Menopausal status | Voorrips et al (2002)  1986-‘92  Netherlands  FFQ  62,573 women & 941 cases  Postmenopausal | 1.16  (0.87-1.56)  p=0.23 | 1.40  (0.97-2.03)  p=0.11 | 0.61  (0.38-0.96)*  p=0.001* | 0.88  (0.65-1.21)  p=0.39 | EPA**^ℓ^** =0.98 (0.72-1.35)  p =0.87 | LA^n^=1.24  (0.91-1.69)  p =0.02 | Age, history of BBD^p^, maternal BC, BC in one or more sisters, age at menarche, age at menopause, OC use, parity, age at first child birth, BMI, education, alcohol use, current cigarette smoking and total energy. |
|  |  |  |  | *Oleic acid:*  0.67  (0.44-1.03)  p=0.001* |  | DHA^m^ =1.00  (0.72-1.37)  p =0.70 | AA^o^=0.99  (0.73-1.34)  p =0.93 |  |
| Author  Year  Country  Data  Cohort size & cases  Menopausal status | Sieri et al (2002)  1987-‘92  Italy  FFQ  3367 women- 56 cases & 214 controls  Postmenopausal | 3.47  (1.43-8.44) ^*^  p=0.005* | 1.12  (0.31-4.04)  p=0.76 | 2.96  (0.70-12.6)  p=0.14 | 2.03  (0.68-6.03)  p=0.20 | ---- | LA=1.39  (0.51-3.8)  p =0.46 | Education, parity and place of birth |
| Author  Year  Country  Data  Cohort size & cases  Menopausal status | Horn Ross et al (2002)  1995-‘98  USA  FFQ  111,526 women & 711 cases  Both | 0.8  (0.60-1.20)  p=0.4 | 0.8  (0.6-1.2)  p=0.2 | *Oleic acid:*  0.9  (0.6-1.2)  p=0.5 | ---- | ---- | LA=0.9  (0.7-1.3)  p =0.9 | Age, daily caloric intake, family history of BC, nulliparity, age at first FTP^q^, age at menarche, physical activity and an interaction for BMI and menopausal status |
| Author    Year  Country  Data  Cases & controls  Menopausal status | Saadatian Elahi et al (2002)  1985-‘98  New York  Serum fatty acid level  197 cases & 197 controls  Both | ---- | 1.46  (0.74-2.83)  p=0.11 | 1.15  (0.6-2.18)  p=0.37 | 0.59  (0.31-1.09)  p=0.09 | ---- | ---- | Age at first full term birth, family history of BC, history of BBD, and total cholesterol |
| Author  Year  Country  Data  Cohort size & cases  Menopausal status | Byrne et al (2002)  1980-‘94  USA  FFQ  44697 women & 1070 cases  Postmenopausal | 0.94^$^  (0.77-1.15)  p=0.57 | 0.88  (0.70-1.12)  p=0.05* | *Oleic acid:*  1.13  (0.81-1.57)  p=0.67 | 0.93  (0.74-1.16)  p=0.75 | ---- | ---- | Age in months, height, age at menarche, combined age at menopause, use of postmenopausal hormones, parity, age at first child birth, BMI at age 18, weight change since age 18, family history of BC, alcohol, vitamin A and total energy intake |
| Author  **Table 2 (Contd….)**  Year  Country  Data  Cohort size & cases  Menopausal status | Velie et al (2000)  1973-‘95  USA  FFQ  40,022 women & 996 cases  Postmenopausal | 1.07  (0.86-1.32)  p=0.51 | 1.12  (0.87-1.45)  p=0.67 | *Oleic acid:*  0.88  (0.62-1.25)  p=0.92 | ---- | ---- | LA=1.05  (0.82-1.34)  p =0.44 | Total energy, BMI, height, parity, family history of BC, age at first child birth, age at menopause, history of BBD, menopausal status, alcohol and education |
| Author  Year  Country  Data  Cohort size & cases  Menopausal status | Holmes et al (1999)  1980-‘94  USA  FFQ  88,795 women & 2956 cases  Pre-menopausal | 0.97  (0.94-1.0) ^*^ | 0.94  (0.88-1.01) | 0.94  (0.88-1.0) ^*^ | 0.91  (0.79-1.04) | ---- | ---- | Age, energy, intake of alcohol and vitamin A, time period, height, parity, age at first child birth, age at menarche, age at menopause, use of HRT, age at first child birth, BMI at age 18, weight change since age 18, family history and BBD |
| Author  Year  Country  Data  Cohort size & cases  Menopausal status | Gaard et al (1995)  1977-‘90  Norway  FFQ  25,892 women & 248 cases  Premenopausal | 1.25  (0.86-1.81)  p=0.18 | (0.75-1.75)  p=0.74 | 1.72  (1.19-2.49) ^*^  p=0.01^*^ | ---- | ---- | ---- | Age, height, BMI, menopausal status, smoking and energy |
| Author  Year  Country  Data  Cohort size & cases  Menopausal status | Toniolo et al (1994)  1985-‘91  New York  FFQ  14,291 women- 180 cases & 829 controls  Both | 1.49  (0.89-2.48)  p=0.09 | 1.47  (0.88-2.46)  p=0.09 | *Oleic acid:*  1.57  (0.90-2.71)  p=0.24 | ---- | ---- | LA  1.13  (0.65-1.98)  p=0.47 | Energy |
| Author    Year  Country  Data  Cohort size & cases  Menopausal status | van den Brandt et al (1993)  1986-‘89  Netherlands  FFQ  1598 sub-cohort members & 439 cases  Postmenopausal | 1.08  (0.73-1.59)  p=0.32 | 1.39  (0.94-2.06)  p=0.049* | 0.75  (0.5-1.12)  p=0.13 | 0.95  (0.64-1.40)  p=0.85 | ---- | ---- | Age, maternal BC, history of BBD, BC in sister(s), age at menarche, age at menopause, OC use, parity, age at first child birth, BMI, education, alcohol use, current cigarette smoking and total energy. |
| Author  **Table 2 (Contd….)**  Year  Country  Data  Cohort size & cases  Menopausal status | Kushi et al (1992)  1986-‘89  USA  FFQ  32080 women & 408 cases  Postmenopausal | 1.38  (0.86-2.2)  p=0.18 | 1.07  (0.68-1.68)  p=0.53 | 1.09  (0.70-1.70)  p=0.63 | 1.49  (1.01-2.20)*  p=0.05* | ---- | ---- | Age, family history of BC, history of BBD, age at menopause,, age at first child birth, BMI at age 18, waist to hip ratio, alcohol use and total energy. |

a. Relative risk, b. Saturated fatty acids, c. Monounsaturated fatty acids, d. Polyunsaturated fatty acids, e. Food frequency questionnaire, f. Oral contraceptive use, g. Body mass index, h. Breast cancer, i. 7-Day food dairy, j..Hormone replacement therapy, k. Diet history method, ℓ. Eicosapentaenoic acid, m. Docosahexaenoic acid,

n.Linoleic acid, o. Arachidonic acid, p. Benign breast disease, q. Full term pregnancy, * Statistically significant at 5% level.

$-Relative risks were estimating among 44,697 postmenopausal women with no reported history of benign breast disease.
